# Principles of peptide selection by the transporter associated with antigen processing

**DOI:** 10.1073/pnas.2320879121

**Published:** 2024-05-28

**Authors:** James Lee, Michael L. Oldham, Victor Manon, Jue Chen

**Affiliations:** ^a^Laboratory of Membrane Biophysics and Biology, The Rockefeller University, New York, NY 10065; ^b^HHMI, Chevy Chase, MD 20815

**Keywords:** antigen presentation, MHC-I, adaptive immunity, transporter associated with antigen processing, ABC transporter

## Abstract

Our ability to fight pathogens relies on major histocompatibility complex class I (MHC-I) molecules presenting diverse antigens on the surface of diseased cells. The transporter associated with antigen processing (TAP) transports nearly the entire repertoire of antigenic peptides into the endoplasmic reticulum for MHC-I loading. How TAP transports peptides specific for MHC-I is unclear. In this study, we used cryo-EM to determine a series of structures of human TAP, both in the absence and presence of peptides with various sequences and lengths. The structures revealed that peptides of eight or nine residues in length bind in a similarly extended conformation, despite having little sequence overlap. We also identified two peptide-anchoring pockets on either side of the transmembrane cavity, each engaging one end of a peptide with primarily main chain atoms. Occupation of both pockets results in a global conformational change in TAP, bringing the two halves of the transporter closer together to prime it for isomerization and ATP hydrolysis. Shorter peptides are able to bind to each pocket separately but are not long enough to bridge the cavity to bind to both simultaneously. Mutations that disrupt hydrogen bonds with the N and C termini of peptides almost abolish MHC-I surface expression. Our findings reveal that TAP functions as a molecular caliper that selects peptides according to length rather than sequence, providing antigen diversity for MHC-I presentation.

## Significance

Cells present peptide antigens on their cell surfaces for immunosurveillance. Peptides destined for display are first delivered into the endoplasmic reticulum (ER) by the transporter associated with antigen processing (TAP). How TAP recognizes and transports a defined but very diverse set of peptide antigens across the ER membrane is a long-standing question in immunology. Through analyzing the structures of TAP in complex with a series of peptides, we uncovered a simple yet elegant principle: TAP grasps each peptide at its two ends, leaving the central positions suspended within a large transmembrane cavity. By prioritizing interactions with main-chain atoms at terminal positions on the peptide, TAP can bind peptides with large variability in sequence and size.

Cytotoxic T cells recognize and eliminate diseased cells upon the detection of foreign peptides displayed by cell-surface MHC-I molecules. The loading of intracellular peptide antigens onto the heterodimeric MHC-I complex is a multistep process (1) that begins when proteins in the cytosol are degraded into small peptides by the proteasome (2, 3). TAP transports a small fraction of these peptides into the endoplasmic reticulum (ER) (4–7). There, the peptide-loading complex selects and loads peptides with high affinity for a specific MHC-I allele to be displayed on the cell surface (8, 9). Peptides derived from endogenous proteins are immunologically silent due to self-tolerance. However, foreign or aberrant peptides resulting from viral infection or malignant transformation trigger an adaptive immune response that eradicates diseased cells.

The sequence space of foreign peptides is vast due to the combined diversity of viral proteomes and products of malignant transformation. MHC-I and TAP have therefore evolved to interact with a broad spectrum of peptides. The principles governing MHC-I presentation are well understood (10–12). Humans have three classical MHC-I genes (HLA-A,B,C) that are highly polymorphic, resulting in vast genetic diversity within a population. Each MHC-I allele is able to present a unique cohort of peptides by forming interactions with their backbone and the side chains of a few specific anchoring residues. In contrast, TAP within each individual is expressed from a single lucus with limited polymorphism (13–17). Although TAP supplies multiple MHC-I alleles with a broad range of peptides, studies using peptide libraries have shown that TAP exercises a certain degree of preference for the length and sequence of substrates. TAP most efficiently transports peptides of 8 to 12 residues in length, similar to the length specificity of MHC-I molecules (18, 19). The upper size limit has not been clearly established, but it is widely accepted that peptide antigens with fewer than eight amino acids are inefficient in competing with model peptides or being translocated into the ER (18, 20–22). A further similarity between the specificity of TAP and MHC-I is that peptides must have free N and C termini to bind—modification of either terminus substantially reduces peptide recognition by TAP (23–26).

However, little is known about the mechanism by which TAP recognizes a defined set of diverse peptides. EPR, NMR, and molecular modeling have suggested that peptides bind in an extended conformation, but the position of the bound peptide remains unclear (27–29). Studies using combinatorial peptide libraries have indicated that the terminal positions of peptides play a significant role in determining binding affinity, but TAP’s sequence preference remains ambiguous (22, 25, 30, 31). Genetic and biochemical analyses have identified residues throughout TAP that are important for substrate binding (32–37), but a specific binding site has not been identified. In the absence of direct structural visualization, the molecular basis of TAP’s broad specificity for peptide antigens remains unclear.

In this study, we analyzed the structure of TAP in the presence and absence of peptides. These structures show that TAP suspends peptides across its transmembrane cavity, anchoring their termini in two distal binding pockets primarily through their backbone atoms. A minimum of eight residues is necessary to bridge and bind both pockets. TAP mutations that disrupt interactions with peptide termini abolish MHC-I assembly, underscoring the importance of terminal hydrogen bonds for peptide transport.

## Results

### Peptide Binding Brings the Nucleotide-Binding Domains Closer.

**Fig. 1.**
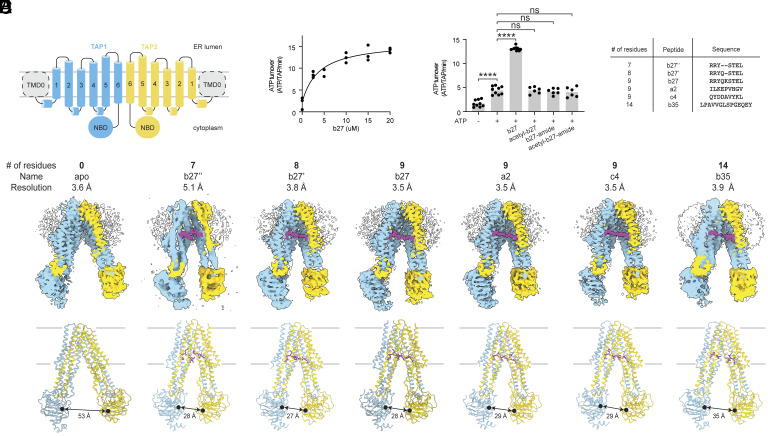
Peptide-binding brings the nucleotide-binding domains together. (*A*) Topology diagram of the TAP complex. TAP1 and TAP2 are colored in cyan and yellow, respectively. (*B*) ATPase activity as a function of b27 concentration. Data represent measurements from three technical replicates (n = 3) collected at 28 °C using 0.5 µM TAP and 3 mM ATP. (*C*) ATPase activity in the presence of b27 variants. Data represent means and SEs from three technical replicates of three biological replicates for −ATP, +ATP, and +ATP/b27 conditions (n = 9) and three technical replicates of two biological replicates for termini-modified peptides (n = 6). Concentrations: TAP, 0.5 µM; peptides, 20 µM; ATP, 3 mM. Statistical significance tested by one-way ANOVA. ns, not significant; ****P < 0.0001. (*D*) Peptides used in this study. (*E*) cryo-EM densities (*Upper*) and ribbon representations (*Lower*) of TAP in the presence and absence of peptide substrates. Densities are sliced perpendicular to the membrane to show the translocation pathway. TAP1, TAP2, and peptides are colored in skyblue, gold, and magenta, respectively. Unassigned densities are colored white. Membrane boundaries are marked by gray lines and the distance between TAP1 D668 and TAP2 E632 are indicated. TAP reconstructions corresponding to apo, b27″, b27′, b27, a2, c4, and b35 are contoured to 0.22, 0.55, 0.20, 0.10, 0.23, 0.20, and 0.14 SDs, respectively.

TAP is a heteromeric ATP-binding cassette (ABC) transporter comprising two subunits, TAP1 and TAP2 (Fig. 1*A*). Each subunit has an N-terminal transmembrane domain (TMD0) that interacts with other components of the larger peptide-loading complex, a six-transmembrane helical domain (TMD) that forms the translocation pathway, and a nucleotide-binding domain (NBD) that hydrolyzes ATP (Fig. 1*A*). The TMD0 domains facilitate MHC-I loading but are not essential for peptide transport (38, 39). To investigate the molecular basis of substrate selectivity by TAP, we purified wild-type (WT) human TAP in the detergent glyco-diosgenin (GDN) following overexpression in human embryonic kidney (HEK) cells (*SI Appendix*, Fig. S1*A*). The biochemical properties of the recombinant protein were assessed using the 9-mer peptide sequence RRYQKSTEL (b27), a well-characterized substrate transported by TAP and presented by HLA-B27 (25, 40). In the absence of b27, TAP hydrolyzed ATP at a specific turnover rate of 2 per minute (Fig. 1 *B* and *C*). ATP hydrolysis was stimulated in a dose-dependent manner by b27 (Fig. 1*B*), but not by N or C termini–modified b27 (Fig. 1*C*), consistent with previous studies (23–26).

**Fig. 2.**
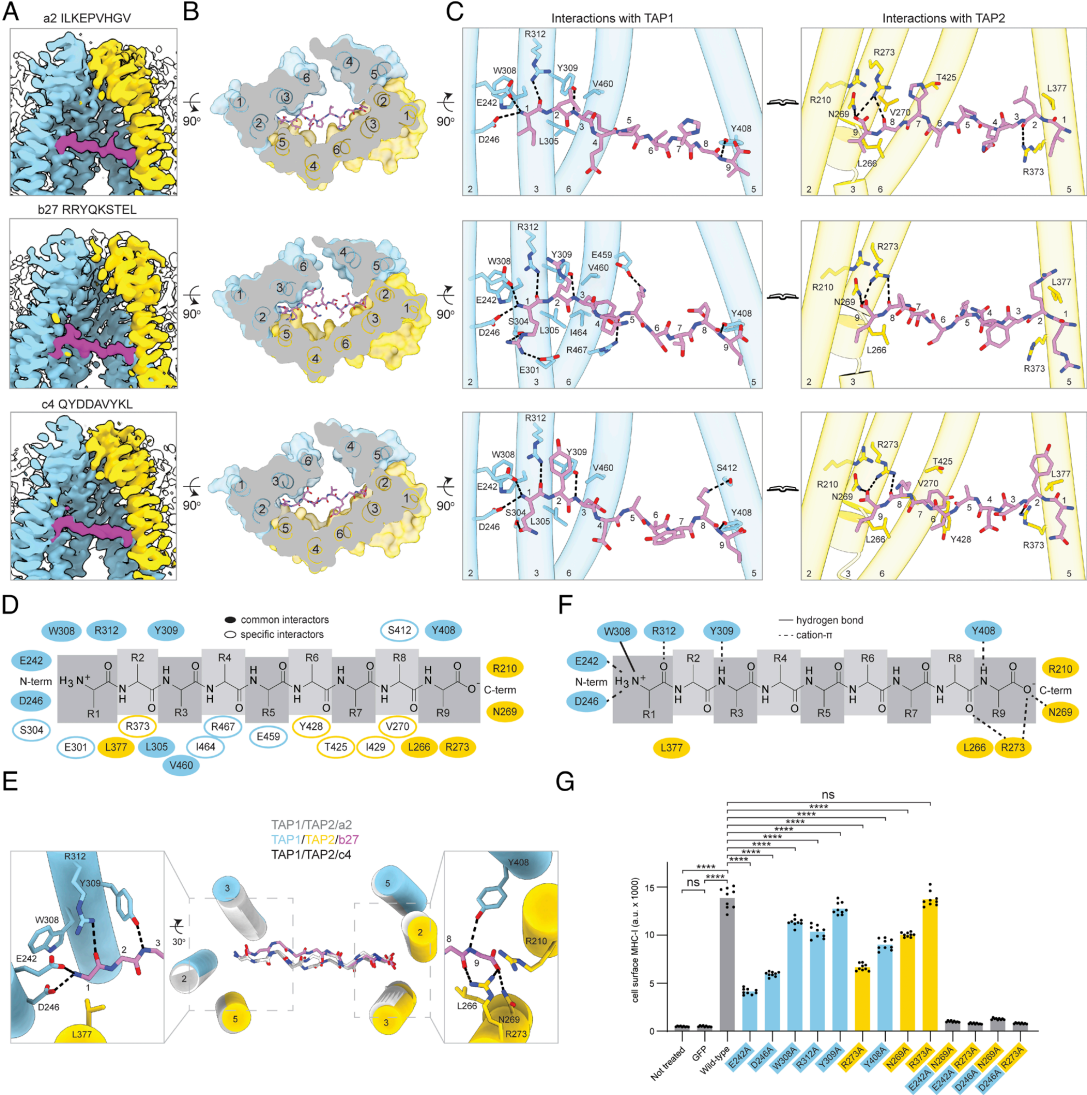
9-mer peptides bind in extended conformations parallel to the membrane. (*A*) Cryo-EM densities of the TAP translocation pathway with bound a2, b27, and c4. Reconstructions are contoured to the same values as in Fig. 1*E*. (*B*) Cross-sectional surface representation of the peptide-binding site viewed from the ER lumen. (*C*) Peptide-binding residues on TAP1 (*Left*) and TAP2 (*Right*). Only transmembrane helices within 4 Å of the peptide are shown. Hydrogen bonds are marked as black dashed lines. (*D*) Schematic of the residues in TAP involved in peptide binding. Residues that interact with all three 9-mer peptides are indicated as filled ovals and those that interact with only 1 or 2 peptides are indicated as open ovals. (*E*) Superposition of the three 9-mer peptides and adjacent transmembrane helices (side chains have been omitted for clarity). *Insets* display conserved interactions at the N and C termini. (*F*) Schematic showing TAP–peptide interactions that were observed for all three 9-mers. (*G*) Flow cytometry analysis of MHC-I cell surface levels in TAP KO cells expressing GFP-tagged TAP variants. Data represent median fluorescence intensity of three technical replicates of three biological replicates (n = 9). Statistical significance tested by one-way ANOVA. ns, not significant; ****P < 0.0001.

We determined the cryo-EM structures of TAP in the absence of peptide (apo) and presence of six different peptides between seven and 14 residues long (Fig. 1 *D* and *E* and *SI Appendix*, Figs. S1–S3). All structures were determined in the absence of ATP, which reduces the affinity of peptides for TAP (41). The highest quality maps were obtained for 9-mer-bound TAPs, followed by apo, 8-mer-, and 14-mer-bound forms. Although the flexibility of the TAP1 NBD reduced the overall resolution, densities for the TMDs and bound peptides were well defined, permitting unambiguous assignment of the molecular moieties forming TAP–substrate interactions (Figs. 1*E* and 2*A* and *SI Appendix*, Figs. S1–S3). The resolution of the 7-mer peptide-bound structure was limited to 5.1 Å due to greater heterogeneity (*SI Appendix*, Fig. S1*F*). In all reconstructions, amorphous densities corresponding to TMD0 domains are visible only at a lower contour, indicating their flexibility. All structures exhibit similar NBD-separated conformations in which the peptide-translocation pathway is open to the cytoplasm (Fig. 1*E*). NBD separation is greatest in the apo structure (53 Å), and reduced in the presence of peptides to between 35 Å (for the 14-mer peptide) and 27 Å (for the 8-mer peptide).

We observed two different TMD configurations among the seven structures (*SI Appendix*, Fig. S4). Five structures have a typical inward-facing conformation with a fully sealed translocation pathway at the ER luminal side (*SI Appendix*, Fig. S4*A*). In contrast, structures obtained with the b27 (9-mer) and b27″ (7-mer) peptides have a lateral opening between the transmembrane (TM) helices that connects the translocation pathway to the membrane (*SI Appendix*, Fig. S4 *B* and *C*). Although this gap could provide access to the ER lumen for bound peptides, it is blocked by densities representing putative lipid molecules in both structures (*SI Appendix*, Fig. S4 *B–D*). Furthermore, we found no correlation between the length or sequence of bound peptide and the occurrence of this lateral opening. A similar lateral opening was observed in a previous structure of TAP stabilized by the viral inhibitor ICP47 (42, 43). Whether this opening is an intrinsic step in the peptide transport cycle, or merely a reflection of structural flexibility in the absence of other members of the peptide-loading complex, remains a subject for future investigation.

### 9-mer Peptides Bind in Extended Conformations Parallel to the Membrane.

To understand how TAP recognizes peptide antigens, we analyzed the structures of TAP in complex with b27 and two other 9-mer peptides, all of which have optimal lengths for binding (Fig. 2) (18, 19). These three peptides, b27, ILKEPVHGV (a2), and QYDDAVYKL (c4), are natural products of the antigen processing pathway and are presented by HLA-B27, HLA-A2, and HLA-C4, respectively (40, 44, 45). The structures of a2- and c4-bound TAP are essentially identical, having an overall rmsd of 0.8 Å. In contrast, the b27-bound structure exhibits a different TMD configuration with a lateral opening as described above (*SI Appendix*, Fig. S4). Nevertheless, the peptides are suspended in a central cavity surrounded by TMs 2-6 from both TAP1 and TAP2 in all three structures (Fig. 2 *A* and *B*). When viewed from a perspective perpendicular to the membrane plane, each peptide stretches roughly 27 Å across the TM cavity (Fig. 2*B*).

Despite their sequence diversity, the three 9-mer peptides are stabilized in the peptide-binding pocket by a common set of interactions involving backbone atoms on each end of the peptide (Fig. 2 *E* and *F*). These interactions, including eight hydrogen bonds, a cation-π, and two van der Waals interactions, enable sequence-independent binding to TAP. At the N-terminal region, the free amino group of each peptide forms a cation-π interaction with TAP1 W308 and two hydrogen bonds with TAP1 E242 and D246 (Fig. 2 *E* and *F*). The carbonyl oxygen and amide at positions one and three are coordinated by hydrogen bonds with TAP1 R312 and Y309, respectively. Furthermore, TAP2 L377 forms van der Waals interactions with the N terminus of all three peptides, and R373 interacts with the main chain atoms of a2 and c4 but not b27 (Fig. 2 *C* and *D*). In comparison, binding of the C-terminal end of each peptide is dominated by interactions with TAP2 (Fig. 2 *E* and *F*). The terminal carboxyl group of all three peptides forms hydrogen bonds with TAP2 N269 and R273, as well as longer-range electrostatic interactions with TAP2 R210. The terminal amide bond on each peptide is stabilized by van der Waals interactions with TAP2 L266, as well as hydrogen bonds with TAP2 R273 and TAP1 Y408. The latter interaction is consistent with mutagenesis studies identifying Y408 as a residue that interacts with the C-terminal end of peptides (14, 46). In addition to these common interactions, side chains on the first three and last amino acids of peptides, as well as occasional side chains in more central positions, make variable contacts with TAP, permitting sequence-dependent enhancement of binding (Fig. 2 *C* and *D*).

All backbone-binding residues on TAP1 and TAP2 are highly conserved among different species (*SI Appendix*, Fig. S5). To investigate their functional significance in a cellular context where peptides with variable length and sequences are available, we analyzed surface expression of MHC-I molecules in cells carrying different TAP variants (Fig. 2*G* and *SI Appendix*, Fig. S6 *A* and *B*). In TAP-knockout (KO) cells, MHC-I surface presentation is severely impaired, consistent with the essential role of TAP in providing peptide antigens to complete MHC-I folding. Although rescue by WT TAP restores MHC-I surface expression, rescue by TAP mutants depends on the position mutated. Alanine mutagenesis of the N-terminal binding residues E242 and D246 in TAP1, and of the C-terminal binding residue R273 in TAP2, resulted in the greatest defects in transport (50 to 60% decrease in surface MHC-I levels relative to WT). In contrast, eliminating the hydrogen bond between TAP1 Y309 and the third peptide backbone amide resulted in a modest reduction (10%). Mutating R373 in TAP2, which interacts with two of the three peptides at position two, had no effect. Strikingly, a combination of substitutions at the N- and C-terminal ends reduced MHC-I surface expression to that in the absence of TAP. This loss of function was not due to reduced expression (*SI Appendix*, Fig. S6*C*) or folding of TAP (*SI Appendix*, Fig. S6 *D* and *E*), indicating that engaging the free amino and carboxyl termini of peptides is critical for TAP to transport substrates.

### The Structure and Electrostatics of Each Anchoring Pocket Confer Sequence Preferences.

**Fig. 3.**
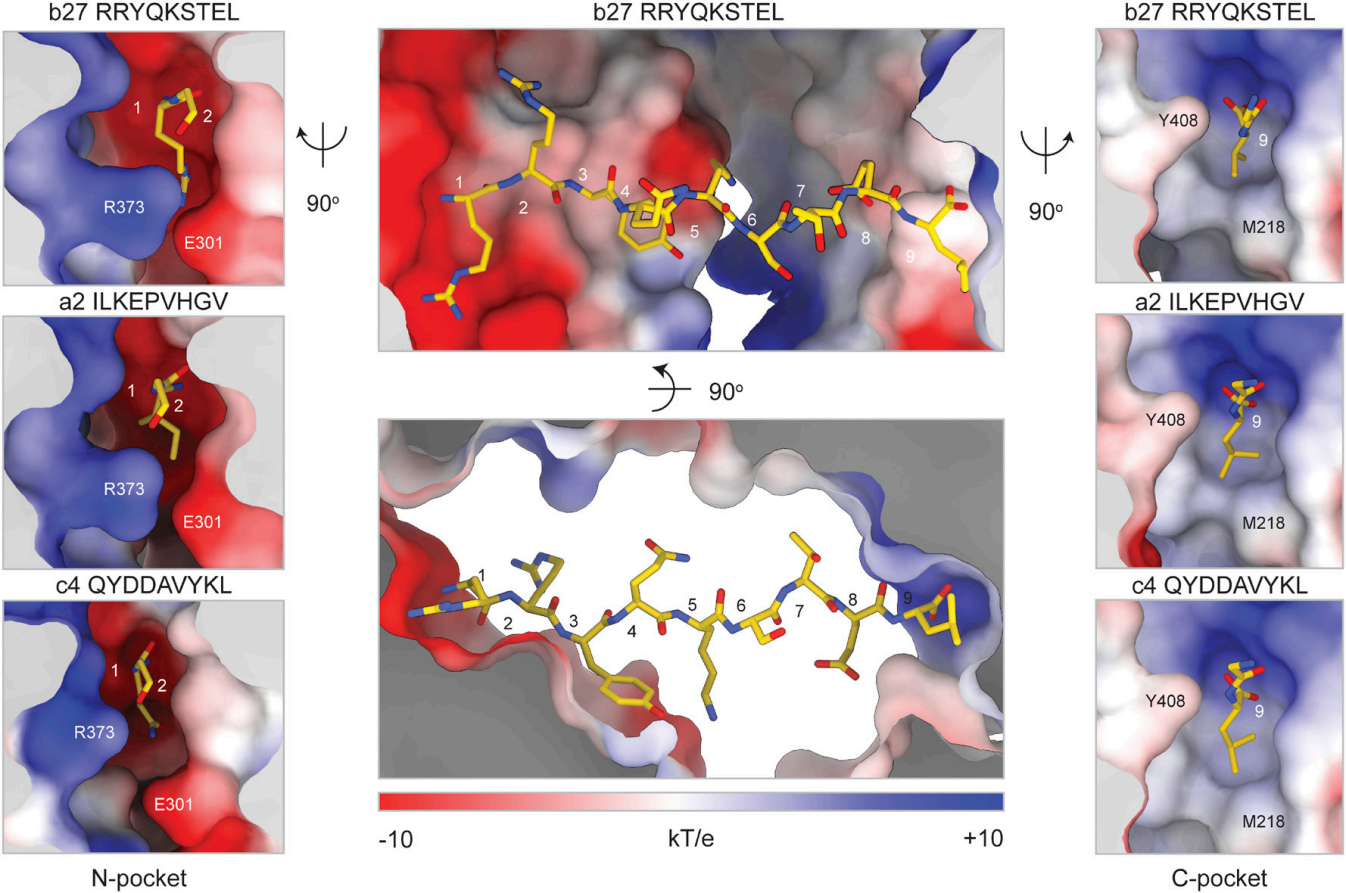
The structure and electrostatics of each binding pocket confer sequence preferences. Electrostatic surface representations of the peptide-binding site. Center, binding of the entire b27 peptide. *Left*, insertion of the first residue of b27 (*Top*), a2 (*Middle*), and c4 (*Bottom*) into the N-pocket. *Right*, binding of the last residue of each peptide to the C-pocket.

Although our data show that TAP primarily binds to the backbones of peptides in a sequence-independent manner, earlier studies demonstrated that TAP exhibits preferences for specific peptide sequences, particularly at their N and C termini (25, 30, 31, 47). We thus considered whether sequence preferences arise from the structural and electrostatic properties of the peptide-binding site (Fig. 3). The amino group of each peptide inserts into a deep pocket (N-pocket) lined with charged and polar residues from TAP1, including E242, D246, W308, and E301 (Figs. 2*D* and 3). The strong negatively charged surface of the N-pocket, together with its narrow entrance formed by TAP1 E301 and TAP2 R373, explain why the free amino terminus of a peptide is important for high-affinity binding, why a positively charged amino acid at position one is preferable (25), and why negatively charged and aromatic residues are disfavored (22, 30) (Fig. 3).

The C terminus of each peptide docks in a pocket (C-pocket) that differs substantially in size, depth, and electrostatic surface potential from the N-pocket (Fig. 3). The C-pocket has two regions; a positively charged surface where the terminal carboxyl group binds and a hydrophobic cleft that interacts with the side chain of the C-terminal residue. Previous studies have shown that peptides with a hydrophobic C-terminal residue have a higher affinity for human, mouse, and rat cim^u^ TAP alleles (20, 23, 24). In contrast, the rat cim^a^ allele is uniquely capable of transporting peptides with a C-terminal basic residue. This functional polymorphism was attributed to a single amino acid difference (*SI Appendix*, Fig. S5). The structures show that in human TAP, M218 lines the hydrophobic region of the C-pocket (Fig. 3), but this residue is replaced by a glutamate in the cim^a^ allele. In this position, E218 would form ionized hydrogen bonds with positively charged side chains on the C termini of peptides, allowing such peptides to be transported.

### At Least Eight Residues Are Required to Engage N- and C-Pockets Concurrently.

**Fig. 4.**
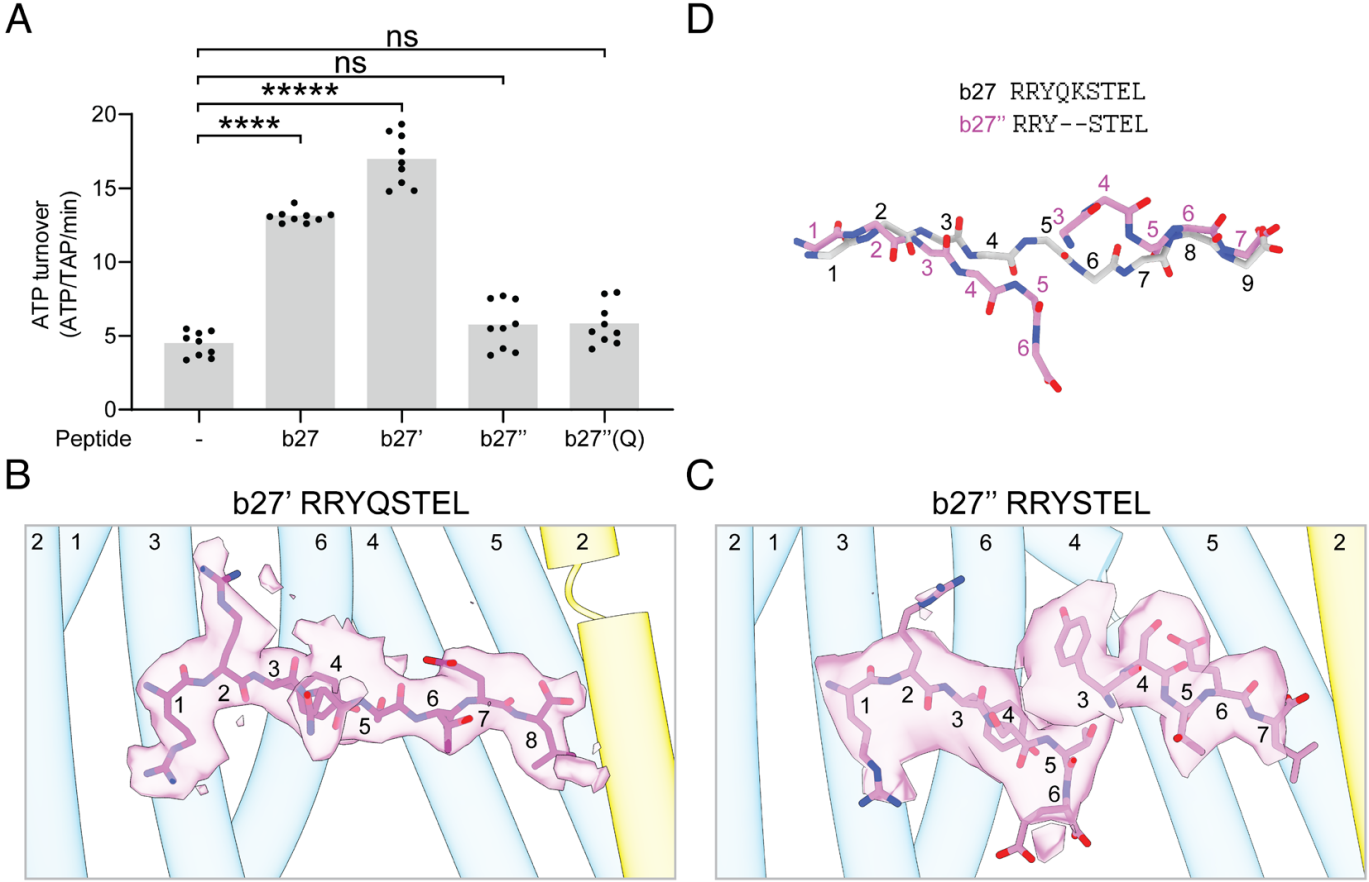
At least eight residues are required to engage N- and C-pockets concurrently. (*A*) The 8-mer (b27′) but not the 7-mer [b27″ and b27″(Q)] peptides stimulated ATP hydrolysis. Data represent means and SEs from three technical replicates of three biological replicates (n = 9). Statistical significance tested by one-way ANOVA. ns, not significant; ****P < 0.0001. (*B* and *C*) EM density for bound 8-mer b27′ (*B*) and 7-mer b27″ (*C*) peptides, shown as transparent magenta surfaces. Density corresponding to b27′ and b27″ are contoured to 0.11 and 0.45 SDs, respectively. (*D*) Overlay of peptide backbones of the 9-mer (gray) and two partially built 7-mer peptides (colored).

It is widely agreed that both TAP and MHC-I are unable to efficiently bind peptides with fewer than eight amino acids (20–22, 48–50). We therefore sought to investigate the molecular basis of TAP’s minimum length requirement by analyzing how it interacts with shorter variations of the b27 peptide (Fig. 4). We created an 8-mer modified b27 peptide (b27′) lacking the central lysine at position five, and two 7-mer modified b27 peptides, each lacking the lysine as well as the adjacent glutamine (b27″) or serine [b27″(Q)]. The 8-mer B27′ increased the ATPase activity of TAP to a slightly greater extend compared to b27 (Fig. 4*A*). In contrast, neither of the 7-mer peptides were able to stimulate ATP hydrolysis, suggesting that their short length compromised their function.

Structural analysis revealed that the N and C termini of b27′ bind to TAP using the same interactions as b27 (Fig. 4*B* and *SI Appendix*, Fig. S7). Because the peptide is shorter than b27 by 2 Å, it induces a minor contraction of the translocation pathway (*SI Appendix*, Fig. S7 *A* and *B*). Intriguingly, the arrangement of the TM helices is more similar to the a2- and c4-bound structures (*SI Appendix*, Fig. S7*A*) than the b27-bound structure (*SI Appendix*, Fig. S7*B*). Consequently, the lateral opening seen in the b27-bound structure is no longer present, and the ER luminal gate is entirely closed (*SI Appendix*, Fig. S7*D*). In contrast, the structure of TAP is heterogenous in the presence of b27″. The final reconstruction, determined at 5 Å resolution, closely resembles that of the b27-bound conformation (Fig. 1*E* and *SI Appendix*, Fig. S7*E*), with lateral openings filled by lipid molecules (*SI Appendix*, Fig. S7*D*). Despite the limited resolution, two distinct peptide densities are apparent within the translocation pathway, each attached to either the N- or C-pockets (Fig. 4*C*). The density in the N-pocket is clear enough to model the first six residues of b27″ (*SI Appendix*, Fig. S7*F*), and the density in the C-pocket can accommodate the last five residues of the peptide (*SI Appendix*, Fig. S7*G*). The first three residues (RRY) in the N-terminal binding site and the last two residues (EL) in the C-terminal binding site bind in the same way as the b27 peptide (Fig. 4*D*); however, the remaining residues of each peptide density diverge. These data suggest either that two 7-mer peptides can bind to TAP at the same time at a concentration of 150 µM or that our reconstruction represents two structures, one with a peptide in the N-pocket and the other with a peptide in the C-pocket. Either way, we infer that peptides with less than eight residues cannot span the transmembrane cavity to engage both binding pockets simultaneously, explaining their lower affinity for TAP and inability to compete with longer peptides.

### TAP Binds Longer Peptides by Allowing Flexibility in Their Central Residues.

**Fig. 5.**
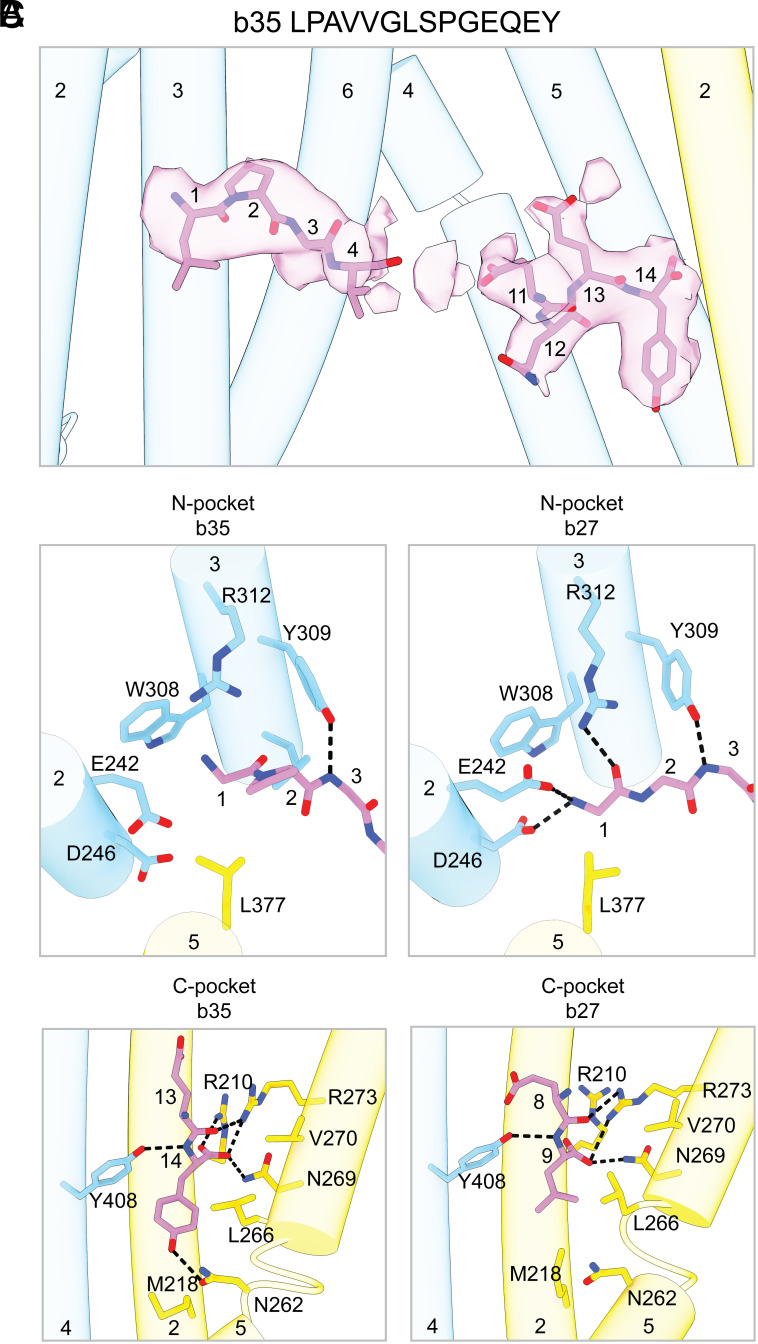
TAP binds longer peptides by allowing flexibility in their central residues. (*A*) Densities corresponding to the 14-mer b35 peptide are shown as transparent magenta surfaces and contoured to 0.14 SDs. (*B*) b35 makes fewer interactions with the N-pocket than the 9-mer b27. The side chains for each peptide have been removed for clarity. (*C*) b35 makes an additional hydrogen bond with the C-pocket compared to b27. Only the last two residues of each peptide are shown. View is from the same perspective as Fig. 3 (*Right*).

Although TAP most efficiently transports peptides between 8 and 12 amino acids in length, the transport of longer peptides has been reported (19, 21, 23). To investigate how TAP interacts with longer peptides, we determined its structure in complex with the 14-mer peptide LPAVVGLSPGEQEY (b35) (Figs. 1*E* and 5 and *SI Appendix*, Fig. S1*K*). b35 contains a proline at position two, which is thought to render it a poor substrate for TAP (51, 52). Nevertheless, b35 is presented on the cell surface by HLA-B35 in a TAP-dependent manner, suggesting it is processed through the canonical antigen processing pathway (53).

The NBDs in the b35-bound TAP structure are separated by a distance intermediate between the apo and 9-mer b27-bound states (Fig. 1*E*). Densities corresponding to the first four and last four residues of the peptide are clearly observed, but the central six residues are largely unresolved (Fig. 5*A*). At lower contour levels, the two densities become continuous and appear linear suggesting that it is one peptide (*SI Appendix*, Fig. S8). Although b35 engages residues in both binding pockets, there are important local differences to the way in which 9-mer peptides bind to TAP (Fig. 5 *B* and *C*). Specifically, the proline at position two gives b35 a different backbone conformation that eliminates the hydrogen bonds with D242, E246, and R312 in the N-pocket (Fig. 5*B*). This corresponds with previous findings that prolines are not well tolerated at position two (25, 52). Conversely, the C-terminal end of b35 makes stronger contacts with TAP compared to the three 9-mer peptides (Fig. 5*C*). The carboxyl group is situated within 3 Å of R210, forming a salt bridge instead of long-range electrostatic interactions. Additionally, the last residue in b35 is a tyrosine instead of a leucine or valine and forms a hydrogen bond with TAP2 N262; a residue known to affect substrate selectivity (36) (Fig. 5*C*). This example provides the structural basis for an earlier proposal that detrimental effects from unfavorable residues can be counterbalanced by favorable residues in other positions (22, 51).

## Discussion

The initiation of a cytotoxic T cell immune response relies on the recognition of foreign peptides, which are presented on the surface of diseased cells by MHC-I molecules. In this study, we sought to understand how these peptide antigens become available for cell-surface presentation by MHC-I. By determining structures of TAP in complex with six different peptides, we demonstrate that TAP grasps each peptide at its two ends, leaving the central positions suspended within a large transmembrane cavity. Interactions primarily occur through hydrogen bonds with the backbone atoms of the peptide, but side chains also play a minor role. By prioritizing interactions with main-chain atoms at terminal positions on the peptide, TAP can bind peptides with large variability in sequence and size. Our data thus reveal a common principle underlying peptide recognition by TAP.

Comparison of the different structures of TAP reveals varying degrees of NBD separation, indicating that the two halves of the transporter can move relative to the central axis (Fig. 1*E*). This flexibility allows TAP to adjust the size of its transmembrane cavity to accommodate peptides of different lengths. The shortest distance we observed between the N- and C-binding pockets was 25 Å in the 8-mer peptide-bound structure. This suggests that a minimum of eight residues is required for a fully extended peptide to be effectively engaged by both binding pockets and initiate high-affinity binding and subsequent transport into the ER lumen. In contrast to longer peptides, which are effectively bivalent ligands for TAP, the affinity of the monovalent 7-mer peptides would be substantially lower. In the cytosol of a cell, where peptides of different lengths are present, 7-mer peptides are unable to compete with longer peptides for binding to TAP. Therefore, the selectivity of transport primarily occurs at the initial binding stage.

The structural data are consistent with many previous studies. Peptide preferences observed in biochemical studies (22, 25, 30, 31) can be explained by the configuration of the binding pockets. The distance between the N and C termini of the bound peptide varies in a narrow range between 25 and 28 Å, comparable to the distances determined by NMR and double electron–electron resonance experiments (27, 29). And finally, many peptide-binding residues, identified through mutagenesis and cross-linking experiments (32–37), are either in direct contact or near the bound peptide.

Four of the peptides used in this study have been cocrystallized with MHC-I (40, 44, 45, 53), providing an opportunity to directly compare their recognition by TAP and MHC-I. Peptides bind to MHC-I in a groove between two α-helices atop a β-sheet (11, 12). Similar to TAP, the interactions are primarily with the main chain atoms of the free N and C termini of the peptide. Like TAP, the two binding sites on MHC-I are separated by ~25 Å (11), establishing a minimum length of eight residues for peptide binding. It is possible that MHC-I molecules have evolved to meet this size requirement because they rely on TAP to supply the repertoire of peptide antigens to the ER.

However, a major difference between MHC-I and TAP is the involvement of peptide side chains in binding. MHC-I makes extensive interactions with side chains on the peptide (11, 12). Indeed, certain side chains serve as anchors by burying deep inside binding pockets that are specific for a particular MHC-I allele. Thus, side chain interactions not only contribute to the overall affinity of binding but also dictate allele-specific selectivity (54–57). In contrast, TAP does not utilize specific anchoring residues. Favorable side chain interactions enhance peptide affinity, but unlike MHC-I, they are not essential for binding.

The common and unique structural features of TAP and MHC-I correlate with their functional properties. Given the challenge of achieving both high affinity and promiscuity simultaneously, TAP has evolved to accommodate sequence diversity and a wide range of peptide affinities (22, 25). In contrast, individual MHC-I molecules prioritize affinity to prevent dissociation of the peptide when positioned on the cell surface. Diversity of peptide presentation is achieved by genetic polymorphism of MHC-I to ensure protection at the population level. Through this delicate balance, TAP provides a comprehensive reservoir of peptides for the numerous MHC-I alleles and each MHC-I binds a specific subset of peptides with high affinity to ensure immune fidelity. Such a coordinated mechanism enables humans to survey a wide range of peptide antigens from an unpredictable and highly variable pathogenic landscape.

## Materials and Methods

### Insect Cell Lines and Culture Conditions.

*Spodoptera frugiperda* Sf9 cells (ATCC CRL-1711) were cultured in Sf-900 II SFM (Gibco) supplemented with 5% (v/v) heat-inactivated fetal bovine serum (FBS) (Gibco), and 1% (v/v) antibiotic–antimycotic (Gibco) at 27 °C.

### Mammalian Cell Lines and Culture Conditions.

HEK293S GnTI- cells (ATCC CRL-3022) were cultured in Freestyle 293 medium (GIBCO) supplemented with 2% (v/v) FBS at 37 °C with 8% CO_2_ and 80% humidity. All cell lines were authenticated by their respective suppliers. Cell lines were tested monthly for mycoplasma contamination by PCR using a Universal Mycoplasma Detection Kit (ATCC) and verified to be negative.

### Protein Expression.

Human TAP was expressed in HEK293S GnTI- cells using an adapted protocol (58). Human TAP1 with a C-terminal PreScission Protease-cleavable GFP tag and TAP2 were cloned into separate BacMam vectors to generate pEG TAP1-GFP and pEG TAP2, respectively. For TAP1/TAP2 complex coexpression, the individual plasmids were combined via SphI and AvrII restriction sites in pEG TAP1-GFP and via SphI and NheI restriction sites in pEG TAP2 to generate pEG TAP1-GFP/TAP2. Bacmid carrying TAP was generated by transforming DH10Bac *Escherichia coli* cells with the pEG TAP1-GFP/TAP2 vector.

Recombinant baculovirus was generated by transfecting Sf9 cells with bacmid using Cellfectin II (Invitrogen). Baculoviruses were harvested from Sf9 cell media by filtering through a 0.22 µm filter and amplified three times before using for cell transduction. Proteins were expressed in HEK293S GnTI- cells infected with 5% (v/v) of baculovirus at a density of 2.5 to 3.0 × 10^6^ cells/mL. Cells were induced with 10 mM sodium butyrate 8 to 12 h after infection and cultured at 30 °C for another 48 h. Cells were harvested, snap frozen in liquid nitrogen, and stored at −80 °C.

### Protein Purification.

Cells were thawed and resuspended in lysis buffer containing 50 mM HEPES (pH 6.5 with KOH), 400 mM KCl, 2 mM MgCl_2_, 1 mM dithiothreitol (DTT), 20% (v/v) glycerol, 1 μg mL^−1^ pepstatin A, 1 μg mL^−1^ leupeptin, 1 μg mL^−1^ aprotinin, 100 μg mL^−1^ soy trypsin inhibitor, 1 mM benzamidine, 1 mM phenylmethylsulfonyl fluoride (PMSF), and 3 µg mL^−1^ DNase I. Cells were lysed by three passes through a high-pressure homogenizer at 15,000 psi (Emulsiflex-C3; Avestin). Unbroken cells and cell debris were removed by one low-speed spin at 4,000 g for 15 min at 4 °C. The supernatant was subjected to a second round of ultracentrifugation at 100,000 × g for 1 h at 4 °C in a Type 45Ti rotor (Beckman) to pellet cell membranes. Membranes were resuspended by manual homogenization in a dounce in lysis buffer supplemented with 1% glyco-diosgenin (GDN) (Anatrace) and incubated for 1 h at 4 °C. The insoluble fraction was removed by centrifugation at 75,000 g for 30 min at 4 °C and the supernatant was applied to NHS-activated Sepharose 4 Fast Flow resin (GE Healthcare) conjugated with GFP nanobody pre-equilibrated in lysis buffer. After 1 h, the resin was washed with 20 column volumes of wash buffer containing 50 mM HEPES (pH 6.5 with KOH), 400 mM KCl, 10% glycerol, 1 mM DTT, and 0.01% GDN. To cleave off the GFP tag, PreScission Protease was added to a final concentration of 0.35 mg mL^−1^ and incubated for 12 h at 4 °C. The cleaved protein was eluted with 5 column volumes of wash buffer and collected by passing through a Glutathione Sepharose 4B resin (Cytiva) to remove the PreScission Protease. The eluate was then concentrated using a 15 mL Amicon spin concentrator with a 100-kDa molecular weight cutoff membrane (Millipore) and purified by size exclusion chromatography (SEC) using a Superose 6 Increase 10/300 column (GE Healthcare) pre-equilibrated with SEC buffer containing 50 mM HEPES (pH 6.5 with KOH), 200 mM KCl, 1 mM DTT, and 0.004% GDN. Peak fractions were pooled using a 4 mL Amicon spin concentrator with a 100-kDa molecular weight cutoff membrane (Millipore) and used immediately for grid preparation or hydrolysis measurements.

### Cryo-EM Grid Preparation and Data Acquisition.

TAP purified from gel filtration was concentrated to ~6 mg mL^−1^ and, where appropriate, incubated with 150 µM of the corresponding peptide on ice for 30 min. Grids were prepared by applying 3.5 µL of this TAP/peptide mixture onto a glow-discharged Quantifoil R0.6/1.0 400 mesh holey carbon Au grid with no wait time. The grids were blotted for 3 s with a blot force of 20 and plunged frozen into liquid ethane using an FEI Mark IV Vitrobot at 6 °C and 100% humidity.

All cryo-EM data were collected using a 300 kV Titan Krios transmission electron microscope equipped with a Gatan K3 Summit direct electron detector. All micrographs were collected using SerialEM (59) in superresolution mode. Data collection parameters for different samples are summarized in *SI Appendix*, Table S1.

### Image Processing.

A similar strategy was employed for cryo-EM data processing for all datasets and a representative flowchart is presented in Extended Fig. 2. Superresolution image stacks were gain-normalized, binned by 2, and corrected for beam-induced motion using MotionCor2 (60). Contrast transfer function parameters were estimated using CTFFIND4 (61). Subsequent data processing for all the datasets followed a similar procedure. In general, particles were autopicked from the motion-corrected micrographs with crYOLO using its general model (62), extracted in RELION (63), and imported into cryoSPARC (64). The picked particles were subjected to multiple rounds of 2D classification, and the resulting particles were subjected to ab initio reconstruction with three classes. One class resembled an empty micelle while the other two classes resembled TAP, but with varying continuous density for one of the NBDs. Nonuniform refinement of the best class with the most complete density for the NBDs resulted in a medium-resolution reconstruction of TAP with well-resolved transmembrane helices and NBD2, but with an invisible NBD1. To improve the density of NBD1, all the particles from 2D classification were subjected to iterative rounds of heterogenous refinement using the best reconstruction and a decoy reconstruction with a disordered NBD1 as input models. The resulting particles that gave reconstructions with a complete NBD1 were then subjected to tandem nonuniform refinement followed by local refinement with a protein mask excluding the micelle. These particles were imported into Relion using the csparc2star.py script (65) and subjected to Bayesian particle polishing (66) and refined again in cryoSPARC. FSC curves were generated in cryoSPARC, and resolutions were reported based on the 0.143 criterion. Masking and B-factor sharpening were determined automatically in cryoSPARC during refinement.

### Model Building and Refinement.

The sharpened and unsharpened maps from local refinement were used for model building. The initial model was obtained by docking individual domains of the published structure of ICP47-inhibited TAP1/TAP2 (42) into the cryo-EM maps using UCSF ChimeraX (67). Models were then manually adjusted using Coot where necessary. The models were then iteratively edited and refined in Coot (68), ISOLDE (69), and PHENIX (70). Regions with poor density were removed such as the TMD0 domains or modeled as polyalanine such as the TAP1 NBD. In the case of the TAP2 NBD, a polyalanine homology model based on the crystal structure of mouse TAP1 NBD was first rigid body fit into the density using ChimeraX, manually inspected, and adjusted where necessary. Then, side chains were manually added and refined in Coot. Residues 632 to 654 and 660 to 666 in the TAP2 NBDs were modeled as polyalanine. The quality of the final models was evaluated by MolProbity (71), model-map correlation coefficients (72), and Q-scores (73). Refinement statistics are summarized in *SI Appendix*, Table S1.

### Analysis of Cellular TAP Levels.

HEK293S GnTI- cells were grown in a 6-well plate and infected with 5% (v/v) baculovirus at 37 °C for 36 h. Cells were harvested by resuspension in 1 mL of buffer containing 50 mM HEPES (pH 8.0 with KOH) and 150 mM KCl and spun down in 1.5 mL tubes for 5 min at 4,000 g at 4 °C. The cell pellets were resuspended in 1 mL of the same buffer supplemented with 1% GDN and incubated for 60 min at 4 °C. Cell lysates were clarified by centrifugation at 20,000 g for 2 × 30 min at 4 °C. Supernatants were immediately used for analysis by fluorescent size exclusion chromatography (FSEC) or SDS-PAGE. FSEC analyses were performed using a Superose 6 10/300 column (GE Healthcare) pre-equilibrated with SEC buffer. SDS-PAGE analyses were performed at 180 V for 75 min using precast 4 to 20% Tris-HCl polyacrylamide gradient gels (ThermoFisher). In-gel fluorescence was detected using a ChemiDoc MP imaging system (Bio-Rad).

### ATP Hydrolysis Measurements.

Steady-state ATP hydrolysis activity was measured using a nicotinamide adenine dinucleotide (NADH)-coupled assay. Purified TAP was diluted to a final concentration of 0.5 µM in freshly prepared reaction buffer containing 50 mM HEPES (pH 8.0 with KOH), 150 mM KCl, 2 mM MgCl_2_, 2 mM DTT, 0.004% (w/v) digitonin, 60 µg mL^−1^ pyruvate kinase (Roche), 32 µg mL^−1^ lactate dehydrogenase (Roche), 9 mM phosphoenolpyruvate and 150 µM NADH. Peptide substrates were added to a final concentration of 20 µM unless specified otherwise. Then, 30 µL aliquots were dispensed into Corning 384-well Black/Clear Flat Bottom Polystyrene NBS Microplates, and reactions were initiated by the addition of 3 mM ATP. NADH consumption was measured by monitoring the rate of NADH fluorescence depletion at λ_ex_ = 340 nm and λ_em_ = 445 nm at 28 °C using an Infinite M1000 microplate reader (Tecan). Data were converted to ATP turnover with an NADH standard curve. Technical replicates were measured in parallel from the same protein preparation on the same day and biological replicates were measured from different protein preparations from different cells on different days.

### Generation of Stable TAP Knockout Cells.

TAP-knockout clonal cells were generated by CRISPR/Cas9-mediated gene editing (74). gRNAs targeting exon 1 of human TAP1 and TAP2 were cloned into the lentiCRISPRv2 vector. The resulting plasmids were transfected into HEK293S GnTI- cells and selected using 1 µg mL^−1^ puromycin for 3 days. Puromycin-resistant cells were screened for loss of cell surface MHC-I by flow cytometry and sorted into single cells. After expansion, clones were genotyped by Sanger sequencing.

### Flow Cytometry Analysis of MHC-I Cell Surface Expression.

MHC-I surface expression was analyzed using an allophycocyanin (APC)-coupled antibody W6/32 (eBioscience), which recognizes an epitope shared among all HLA-A,B,C molecules. HEK293S GnTI- cells grown in a 96-well plate were infected with 5% P1 baculovirus encoding wild-type TAP1-GFP/TAP2, a variant, or GFP alone at 37 °C for 36 h. Cells were resuspended in 100 µL FACS blocking buffer [phosphate-buffered saline (PBS) supplemented with 5% (w/v) bovine serum albumin (BSA) (Sigma)] and centrifuged at 400 g for 5 min at 25 °C. Cells were resuspended in FACS blocking buffer supplemented with antibody added at 5 µg mL^−1^ and incubated for 30 min at 4 °C in the dark. Subsequently, the cells were washed two times with FACS blocking buffer. The resulting cell pellets were resuspended in FACS buffer (PBS supplemented with 0.5% (w/v) BSA and 0.1% (w/v) sodium azide and counted using an Attune NxT Flow Cytometer (ThermoFisher). Gating for live cells with moderate levels of the GFP was used to compare MHC-I expression. Data were analyzed using FCS Express (De Novo Software). Technical replicates were measured in parallel from different cells on the same day and biological replicates were measured from different cells on different days.

### Data Quantification and Statistical Analysis.

All the details of the data and statistical analysis can be found in the figure legends and methods. All data values are presented as mean and SEs. Statistical significance was assessed by a one-way ANOVA relative to the wild-type using GraphPad Prism 9.

### Figure Preparation.

Cryo-EM maps and atomic model depictions were generated using UCSF ChimeraX (67). Maps colored by local resolution were generated using cryoSPARC. Multiple sequence alignments were generated using Clustal Omega (75). Surface interface analysis was performed using PDBePISA (76). Graphs and associated statistics were prepared using GraphPad Prism 9. Software used in this project was managed by SBGrid (77). All figures were prepared using Adobe Illustrator.

## Supplementary Material

Appendix 01 (PDF)

## Data Availability

Cryo-EM density maps have been deposited in the Electron Microscopy Data Bank under the accession codes EMDB-41021, (78) EMDB-41028, (79) EMDB-41029, (80) EMDB-41030, (81) EMDB-41031, (82) EMDB-41032, (83) and EMDB-41033. (84) The corresponding atomic models have been deposited in the Protein Data Bank under the accession codes 8T46, (85) 8T4E, (86) 8T4F, (87) 8T4G, (88) 8T4H, (89) 8T4I, (90) and 8T4J. (91) The raw micrographs for all datasets have been deposited into the Electron Microscopy Public Image Archive under the accession codes EMPIAR-11997 (92), EMPIAR-11998 (93), EMPIAR-12009 (94), EMPIAR-11996 (95), EMPIAR-12000 (96), EMPIAR-11999 (97), and EMPIAR-12001 (98). All other data are included in the manuscript and/or *SI Appendix*.
