# Supplementary material for: Principles of peptide selection by the transporter associated with antigen processing
Source: Proc Natl Acad Sci U S A. 2024 May 28;121(23):e2320879121. doi: 10.1073/pnas.2320879121 (PMC11161800; doi:10.1073/pnas.2320879121)
Supplement: Supplementary file 1 — Appendix 01 (PDF) [file pnas.2320879121.sapp.pdf]

Supporting Information for

Principles of peptide selection by the transporter associated with antigen processing

James Lee[1,2], Michael L. Oldham[1,2,3], Victor Manon[1], and Jue Chen[1,2,*]

Jue Chen
Email: juechen@rockefeller.edu

This PDF file includes:

Figures S1 to S8
Table S1

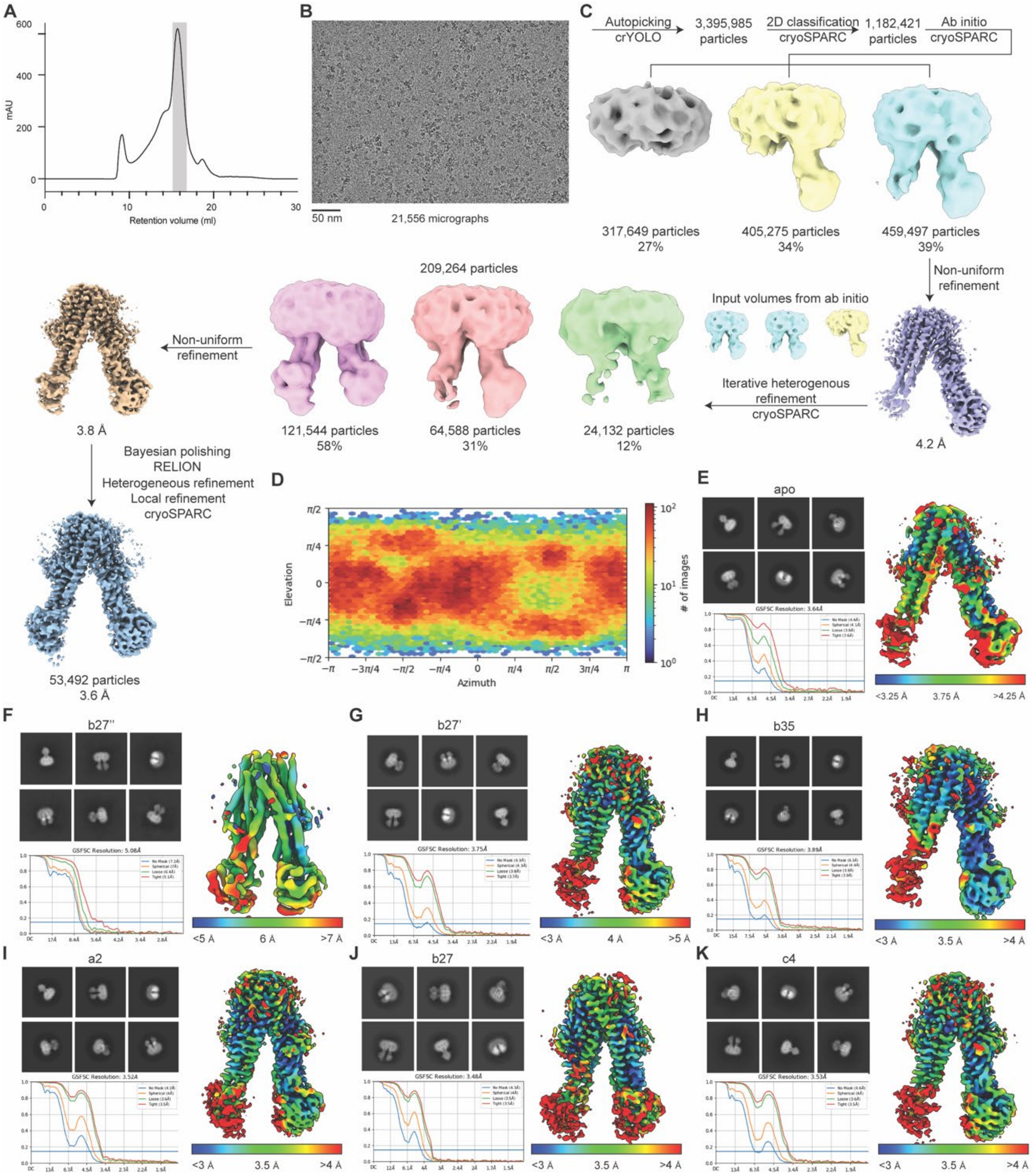

Fig. S1 Summary of structural determination
(**A**) Representative size exclusion profile of purified TAP. Fractions collected for cryo-EM and ATPase assays are marked in grey. (**B**) Representative micrograph of apo TAP. (**C)** Representative flow chart for cryo-EM data processing of apo TAP. (**D**) Angular distribution plot for the final map from cryoSPARC. (**E-K**) Representative 2D classes, the Fourier shell correlation (FSC) curve, colored local resolution maps, and cryo-EM density corresponding to each peptide substrate of the seven structures determined in this study.

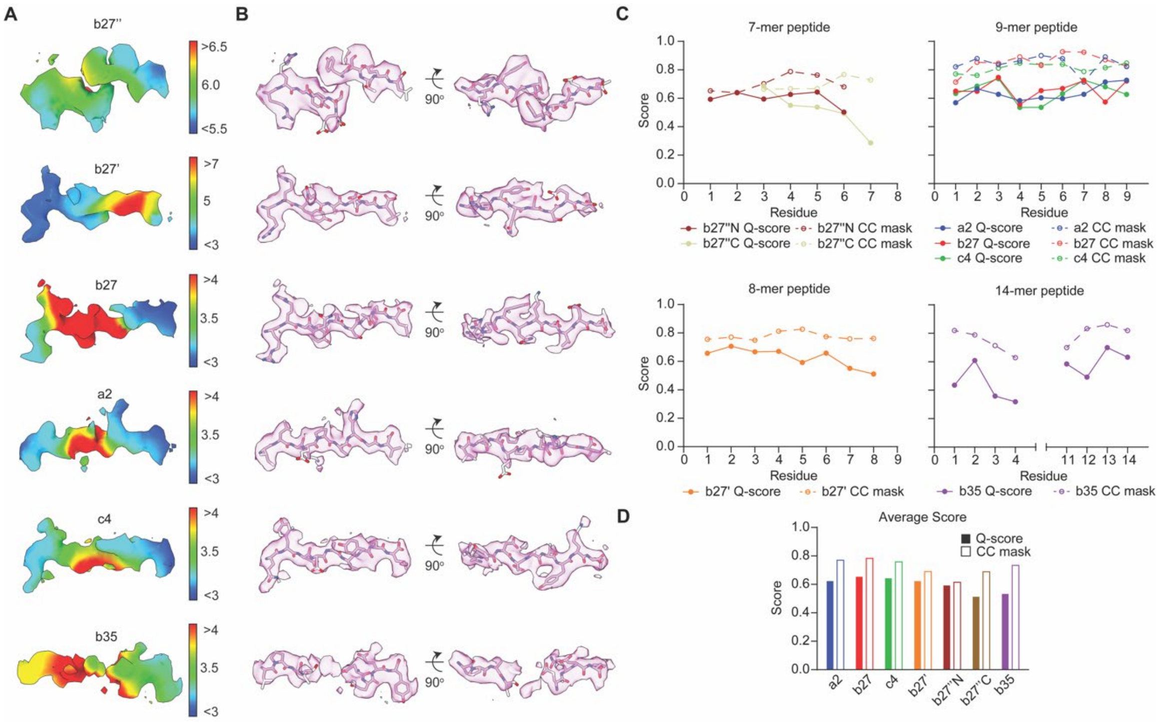

Fig. S2. Quality of the peptide substrate cryo-EM density.
(A) Colored local resolution maps. **(B)** Map to model fit of the peptide substrate. The density corresponding to b27'', b27' b27, a2, c4, and b35 are contoured to 0.45, 0.125, 0.075, 0.125, 0.125, and 0.125 standard deviations, respectively. **(C-D)** Per-residue **(C)** and full peptide **(D)** model-map correlation coefficient and Q-scores. b27''N and b27''C specify the 7-mer peptide that occupies the N- and C-pocket, respectively.

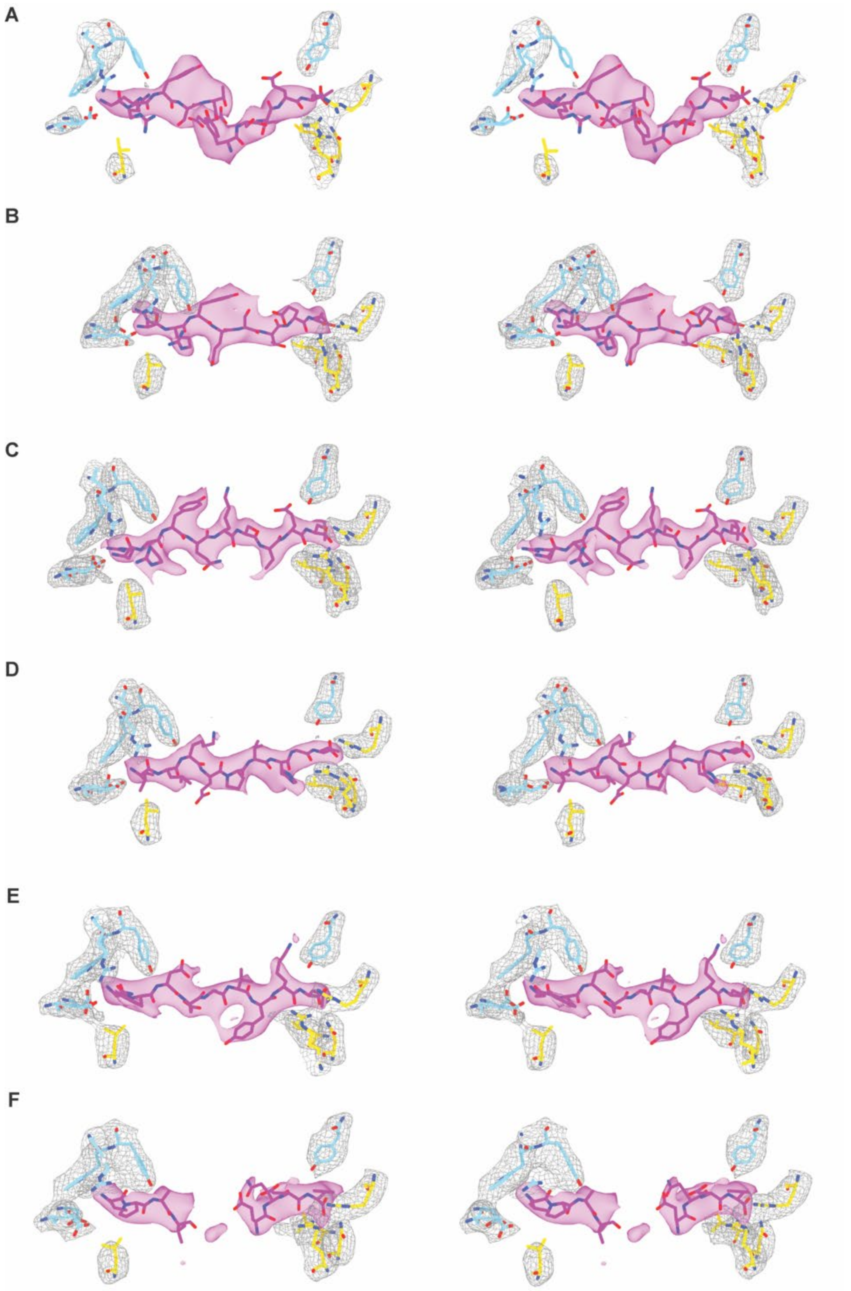

Fig. S3. Stereo views of the peptide substrate binding site cryo-EM density.
(A-F) Density around the substrate binding site of the b27'' **(A)**, b27' **(B)**, b27 **(C)**, a2 **(D)**, c4 **(E)**, and b35 **(F)** peptides as viewed from the ER lumen in Figure 2E. TAP1 E242, D246, W308, Y309, R312, and Y408 along with TAP2 R210, L266, N269, R273, and L377 are shown as sticks. Molecular models of TAP1, TAP2, and the peptide are colored in sky blue, gold, and magenta, respectively. The density of TAP is shown in silver mesh while the density of the peptide is shown in magenta surface. The density corresponding to b27'', b27' b27, a2, c4, and b35 are contoured to 0.45, 0.125, 0.075, 0.125, 0.125, and 0.125 standard deviations, respectively.

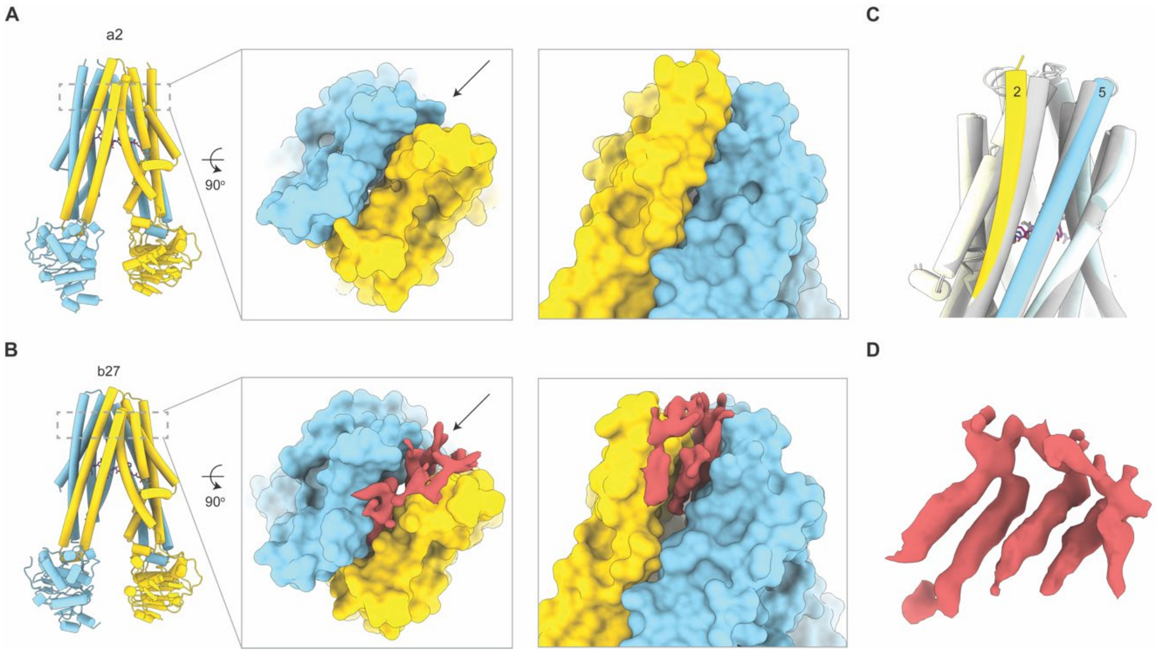

Fig. S4. Two different TMD configurations
(**A**) The structure of a2-bound TAP exhibits a typical inward-facing conformation where the luminal gate (marked by an arrow) is closed. (**B**) The b27-bound TAP opens to the ER lumen through a lateral gate. (**C**) Superposition of the structures of a2 (silver)- and b27 (colored)-bound TAP. (**D**) Densities corresponding to lipids (red) fill the luminal gate in the b27-bound structure.

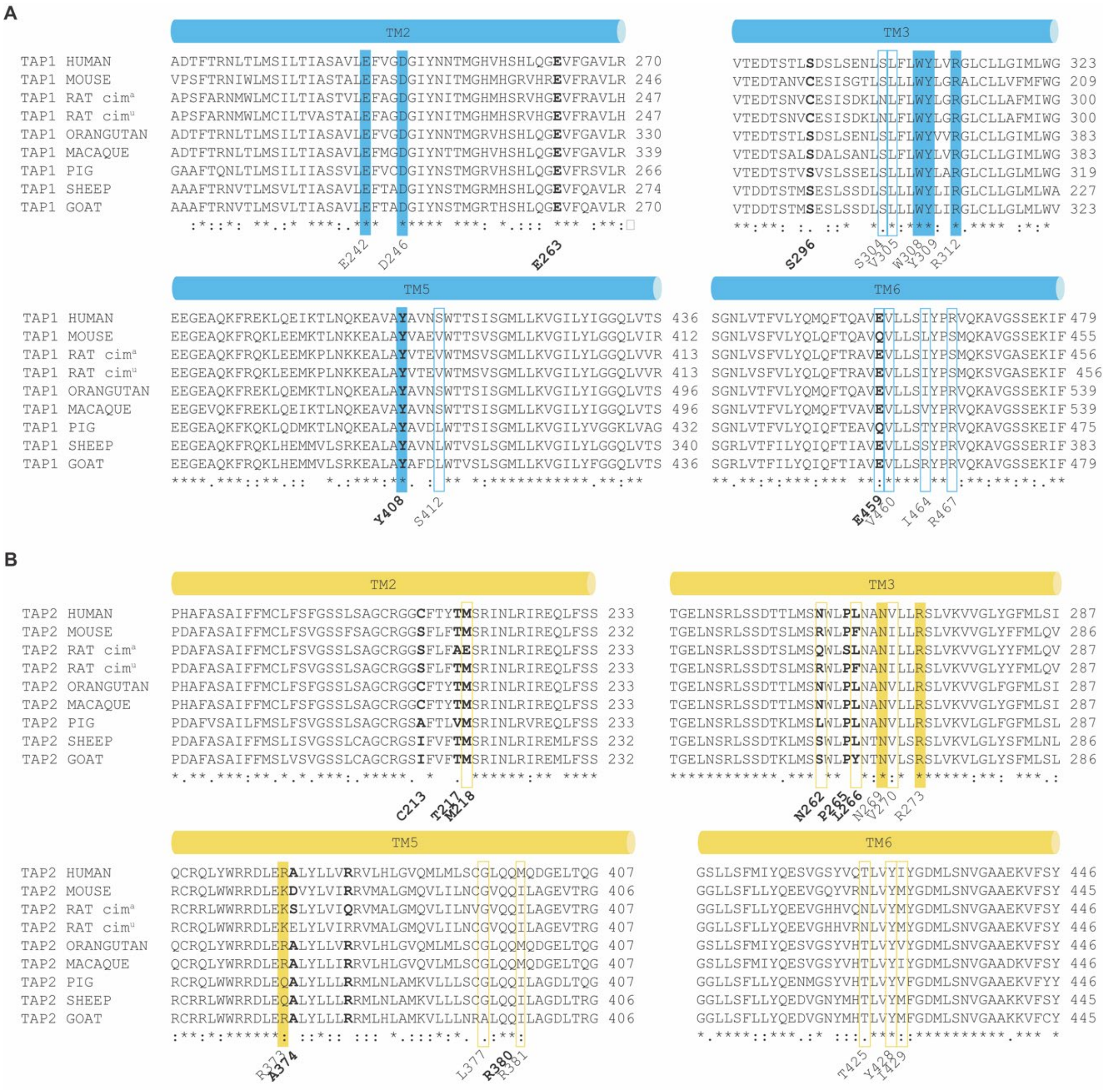

Fig. S5. Sequence alignment of TAP

Sequence alignment of (A) TAP1 and (B) TAP2. Residues identified in this study implicated in peptide binding with either the peptide backbone or peptide side chains are in solid color or outlined, respectively. Residues previously implicated in peptide binding are shown in bold.

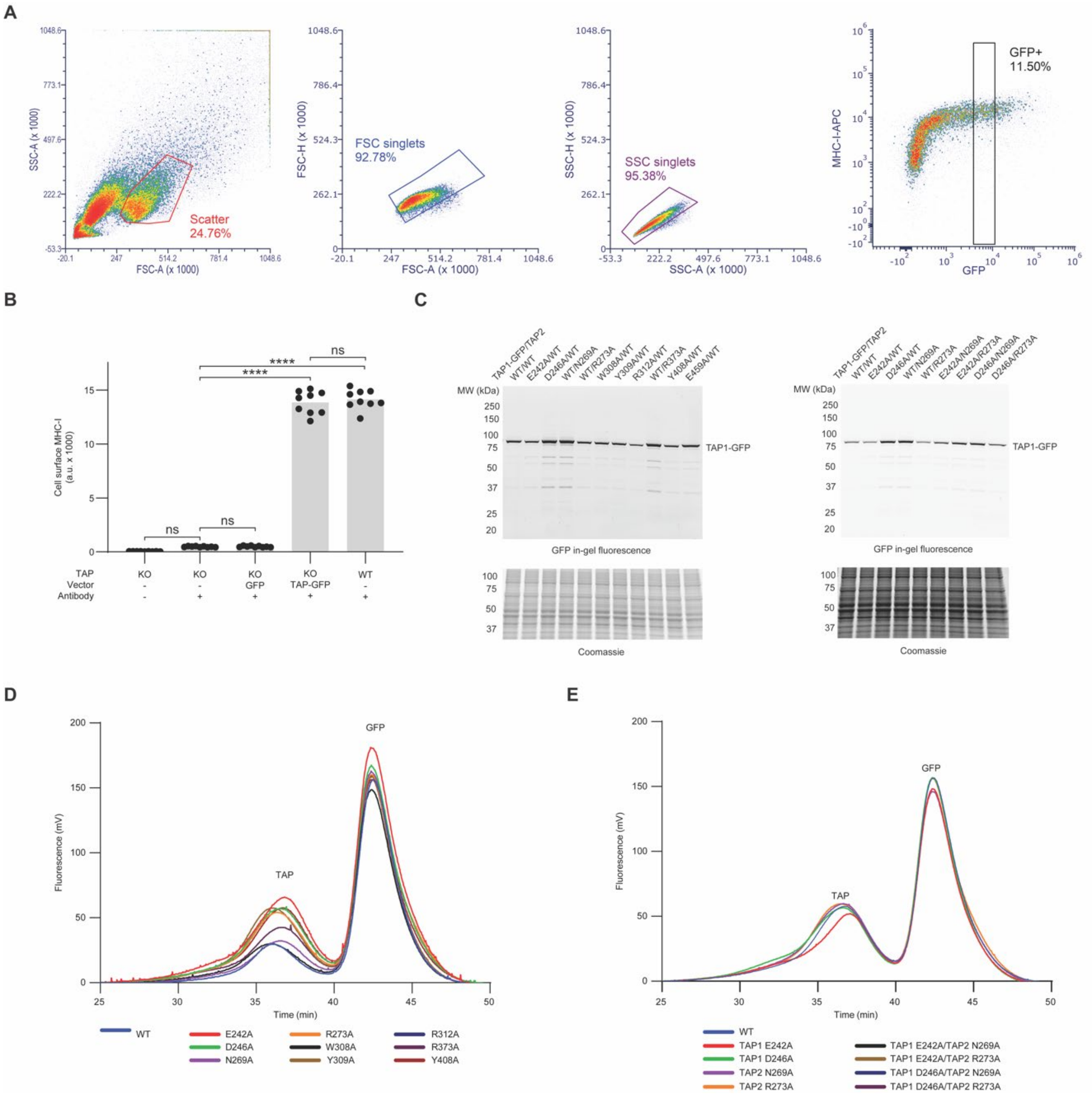

Fig. S6. Characterization of the TAP binding site variants
(A) Representative gating strategy for flow cytometry analysis. Single cells were gated by forward and side scatter. (**B**) Flow cytometry analysis of the TAP KO cell lines. Data represents the means and standard errors from measurements of 3 technical replicates of 3 biological replicates (n = 9). KO, KO GFP, and KO TAP-GFP samples are reproduced from Fig. 2G. (**C**) SDS-PAGE of lysates of cells expressing different GFP-tagged TAP variants visualized by (top) in-gel fluorescence and (bottom) Coomassie stain. (**D-E**) Fluorescence size exclusion chromatography from lysates of cells expressing different GFP-tagged variants. WT, TAP1 E242A, TAP1 D246A, TAP2 N269A, TAP2 R273A samples in (E) are reproduced from (D).

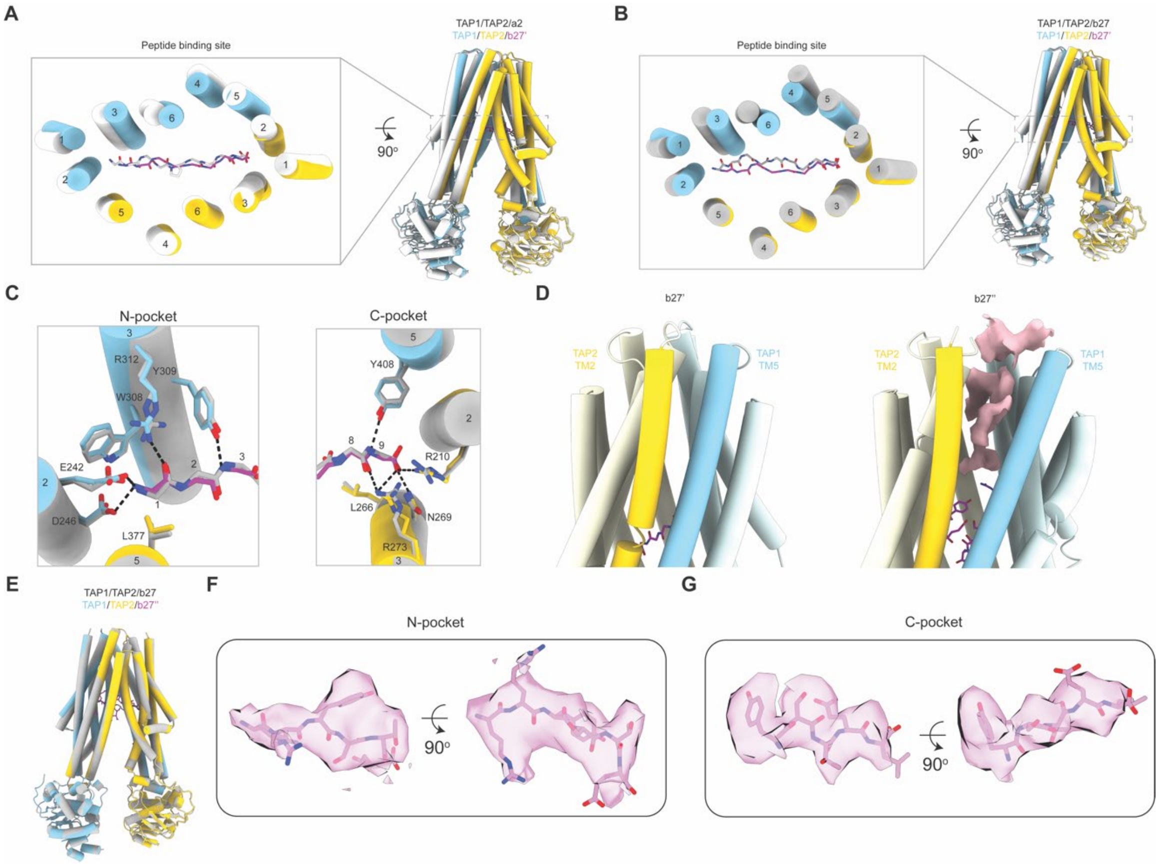

Fig. S7. Structural features in the presence of shorter peptides
(**A-B**) Superposition of the 8-mer b27'-bound structure with the (A) a2- or (B) b27- bound structures. (**C**) Comparison of local conformation of the b27- and b27'- bound structures. The N-pocket (left) is aligned to TM2 and TM3 of TAP1 and the C-pocket (right) is aligned to TM2 and TM3 of TAP2. (**D**) The luminal gate is closed in the b27'-bound structure, but open in the b27''-bound conformation. (**E**) Superposition of the 9-mer and 7-mer-bound structures. (**F-G**) Cryo-EM density corresponding to the N-terminally (F) and C-terminally (G) bound 7-mer peptides.

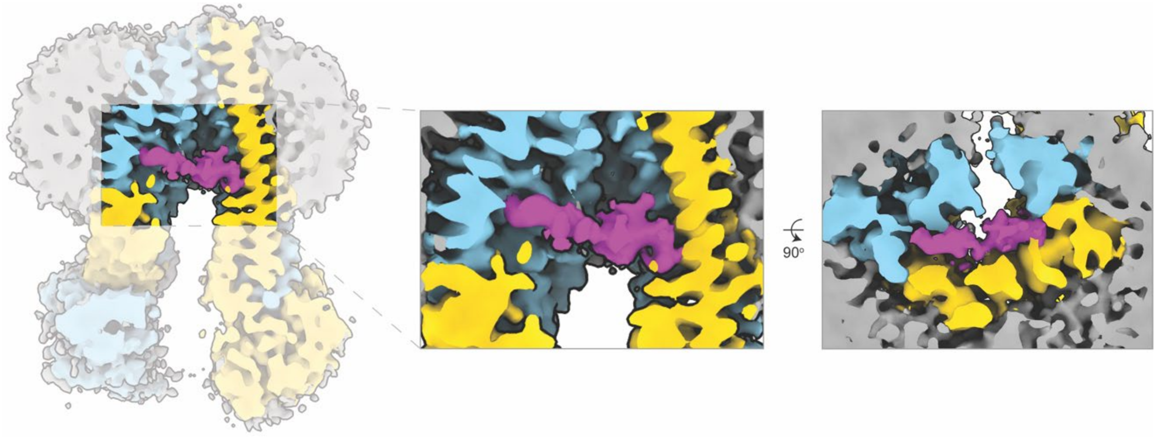

Fig. S8. A single 14-mer peptide likely binds within the TAP translocation pathway.
Cryo-EM reconstruction of TAP bound to the 14-mer b35 peptide at a contour level of 0.1 standard deviations. TAP1, TAP2 and the 14-mer b35 peptide are colored in sky blue, gold and magenta, respectively. Unassigned density is colored silver.

Table S1. Cryo-EM data collection, refinement, and validation statistics, related to STAR Methods

	Apo TAP<br><br>EMDB-41021<br>PDB 8T46	TAP<br>a2 peptide<br>EMDB-41028<br>PDB 8T4E	TAP<br>b27 peptide<br>EMDB-41029<br>PDB 8T4F	TAP<br>c4 peptide<br>EMDB-41030<br>PDB 8T4G
**Data collection and processing**				
Magnification	105,000	105,000	105,000	105,000
Voltage (kV)	300	300	300	300
Electron exposure (e–/Å^2)	50	50	66	50
Defocus range (μm)	0.8 to 2.5	0.8 to 2.5	0.8 to 2.5	0.8 to 2.5
Pixel size (Å)	0.839	0.839	0.676	0.839
Symmetry imposed	C1	C1	C1	C1
Initial particle images (no.)	3,395,985	2,165,210	1,618,719	2,214,564
Final particle images (no.)	79,724	95,683	73,744	48,686
Map resolution (Å)	3.6	3.5	3.5	3.5
FSC threshold	0.143	0.143	0.143	0.143
**Refinement**				
Initial model used (PDB code)	5U1D	8T4F	5U1D	8T4E
Model resolution (Å)	3.8	3.7	3.5	3.6
FSC threshold	0.5	0.5	0.5	0.5
Map sharpening *B* factor (Å^2)	111.6	82.5	83.6	70.6
Model composition				
Non-hydrogen atoms	7886	7714	7794	7804
Protein residues	1113	1089	1079	1108
B factors (Å^2)				
Protein	68.82	75.79	47.11	69.83
R.m.s. deviations				
Bond lengths (Å)	0.003	0.003	0.003	0.003
Bond angles (°)	0.633	0.655	0.571	0.534
Validation				
MolProbity score	1.29	1.44	1.12	1.34
Clashscore	4.88	5.50	3.32	5.37
Poor rotamers (%)	0.28	0.28	0.56	0.14
Model vs Data				
CC (mask)	0.84	0.83	0.79	0.82
CC (box)	0.70	0.69	0.71	0.71
CC (peaks)	0.59	0.57	0.65	0.65
CC (volume)	0.82	0.81	0.77	0.79
Ramachandran plot				
Favored (%)	97.83	97.22	98.34	97.78
Allowed (%)	2.08	2.78	1.66	2.22
Outliers (%)	0.09	0.00	0.00	0.00

Table S1. Cryo-EM data collection, refinement, and validation statistics (cont.)

	TAP<br>b27'' peptide<br>EMDB-41032<br>PDB 8T4I	TAP<br>b27' peptide<br>EMDB-41031<br>PDB 8T4H	TAP<br>B35 peptide<br>EMDB-41033<br>PDB 8T4J
**Data collection and processing**			
Magnification	105,000	105,000	105,000
Voltage (kV)	300	300	300
Electron exposure (e^{-}/Å^{2})	50	50	50
Defocus range (μm)	0.8 to 2.5	0.8 to 2.5	0.8 to 2.5
Pixel size (Å)	0.839	0.839	0.839
Symmetry imposed	C1	C1	C1
Initial particle images (no.)	1,849,805	2,301,048	1,317,654
Final particle images (no.)	24,447	35,603	69.408
Map resolution (Å)	5.1	3.8	3.9
FSC threshold	0.143	0.143	0.143
**Refinement**			
Initial model used (PDB code)	8T4F	8T4E	8T46
Model resolution (Å)	7.4	3.4	4.1
FSC threshold	0.5	0.5	0.5
Map sharpening *B* factor (Å^{2})	418.2	73.0	59.6
Model composition			
Non-hydrogen atoms	7811	7703	7825
Protein residues	1099	1088	1107
B factors (Å^{2})			
Protein	47.72	83.98	99.2
R.m.s. deviations			
Bond lengths (Å)	0.004	0.004	0.002
Bond angles (°)	0.610	0.760	0.600
Validation			
MolProbity score	1.13	1.35	1.27
Clashscore	3.38	5.59	5.04
Poor rotamers (%)	0.84	0.57	0.28
Model vs Data			
CC (mask)	0.65	0.81	0.83
CC (box)	0.59	0.69	0.69
CC (peaks)	0.41	0.55	0.53
CC (volume)	0.64	0.80	0.82
Ramachandran plot			
Favored (%)	98.34	97.77	98.17
Allowed (%)	1.66	2.23	1.83
Disallowed (%)	0.00	0.00	0.00
